# Synovial Chondromatosis and Chondrosarcoma: A Diagnostic Dilemma

**DOI:** 10.1080/13577140310001607293

**Published:** 2003-06

**Authors:** Brita L. Sperling, Steven Angel, Grant Stoneham, Vance Chow, Andrew McFadden, Rajni Chibbar

**Affiliations:** 1 Department of Pathology Royal University Hospital Saskatchewan Saskatoon S7N OW8 Canada; 2 Department of Radiology Royal University Hospital Saskatchewan Saskatoon Canada; 3 Department of General Surgery Royal University Hospital Saskatchewan Saskatoon Canada

## Abstract

*Purpose*: The progression of synovial chondromatosis to chondrosarcoma is very rare. Distinction between these two entities may be difficult on histology alone, and should be based on clinical, radiographic and microscopic evidence. Immunohistochemical markers that would facilitate differentiation between synovial chondromatosis and chondrosarcoma are currently being investigated.

*Patients*: We describe the cases of two patients who presented with synovial chondromatosis and progression to synovial chondrosarcoma during periods of 7 and 11 years. Several biopsies and resected specimens demonstrated synovial chondromatosis before a diagnosis of chondrosarcoma was made.

*Method*: We have examined five markers (Bcl2, Ki67, p27, p16, and p53) in all specimens from these cases, as well as known cases of chondromatosis and chondrosarcoma for control purposes.

*Results*: We found increased expression of Bcl2 in benign chondromatosis compared to synovial or central chondrosarcomas.

*Discussion*: Distinction between chondromatosis and its progression to low grade chondrosarcoma is difficult at histological level, and must involve incorporation of clinical and radiographical data. Although preliminary, our study suggests that reduced or absent expression of Bcl2 is associated withmalignant transformation of chondromatosis.


*Sarcoma*, June 2003, *VOL. 7, NO. 2, 69–73*

Taylor & Francis healthsciences

ORIGINAL ARTICLE

### Synovial chondromatosis and chondrosarcoma: a diagnostic dilemma

BRITA L. SPERLING[1], STEVEN ANGEL[1], GRANT STONEHAM[2], VANCE CHOW[2], ANDREW MCFADDEN[3] & RAJNI CHIBBAR[1]

*Departments of [1]Pathology, [2]Radiology and [3]General Surgery, University of Royal, University Hospital, Saskatoon, Saskatchewan, Canada*

**Abstract**

*Purpose*: The progression of synovial chondromatosis to chondrosarcoma is very rare. Distinction between these two entities may be difficult on histology alone, and should be based on clinical, radiographic and microscopic evidence. Immunohistochemical markers that would facilitate differentiation between synovial chondromatosis and chondrosarcoma are currently being investigated.

*Patients*: We describe the cases of two patients who presented with synovial chondromatosis and progression to synovial chondrosarcoma during periods of 7 and 11 years. Several biopsies and resected specimens demonstrated synovial chondromatosis before a diagnosis of chondrosarcoma was made.

*Method*: We have examined five markers (Bcl2, Ki67, p27, p16, and p53) in all specimens from these cases, as well as known cases of chondromatosis and chondrosarcoma for control purposes.

*Results*: We found increased expression of Bcl2 in benign chondromatosis compared to synovial or central chondrosarcomas.

*Discussion*: Distinction between chondromatosis and its progression to low grade chondrosarcoma is difficult at histological level, and must involve incorporation of clinical and radiographical data. Although preliminary, our study suggests that reduced or absent expression of Bcl2 is associated with malignant transformation of chondromatosis.

**Key words:** *synovial chondromatosis, synovial chondrosarcoma, pathology, radiology, malignant transformation*

### Introduction

Synovial chondromatosis, also known as synovial osteochondromatosis or synovial chondrometaplasia, is an idiopathic synovial proliferation. It is characterized by multiple nodules of metaplastic hyaline cartilage within the synovial membrane of a joint, often detaching to form intra-articular loose bodies.[1] Synovial chondrosarcoma is an uncommon malignant cartilaginous neoplasm arising in synovial tissue, with only 34 reported cases in the literature to date.[2] Most show evidence of concurrent and most likely pre-existing primary synovial chondromatosis, suggesting malignant transformation.[1]

Documented malignant transformation of synovial chondromatosis to chondrosarcoma is quite rare, with approximately 20 cases reported in the literature to date. The risk of progression to malignancy is reportedly as high as 5%.[3]

### Patients

In 1991, a 55-year-old female presented with history of progressive pain in her right hip. Plain radiography of the pelvis and right hip was suggestive of synovial chondromatosis (Fig. 1a). In 1992, the patient underwent synovectomy and removal of clusters of cartilaginous tissue, 3–4 cm in size, the pathology of which was determined to be synovial chondromatosis (Fig. 2a).

This resection provided temporary relief of symptoms. The pain then worsened, necessitating another resection in July 1994. Multiple cartilaginous nodules were excised, and this was followed with an arthroplasty of the right hip. The initial pathology report suggested a low grade chondrosarcoma, but an external consultation with a bone and soft tissue pathologist diagnosed synovial chondromatosis (Fig. 2b).

Correspondence to: Rajni Chibbar, MD, FRCPC, Department of Pathology, University of Saskatchewan, 103 Hospital Drive, Royal University Hospital, Saskatoon, Saskatchewan, SK, Canada S7N OW8. Tel.: +1-306-655-2153. Fax: +1-306-655-2223. E-mail: chibbarr@sdh.sk.ca

ISSN 1357-714X print/1369–1643 © 2003 Taylor & Francis Ltd
DOI: 10.1080/13577140310001607293

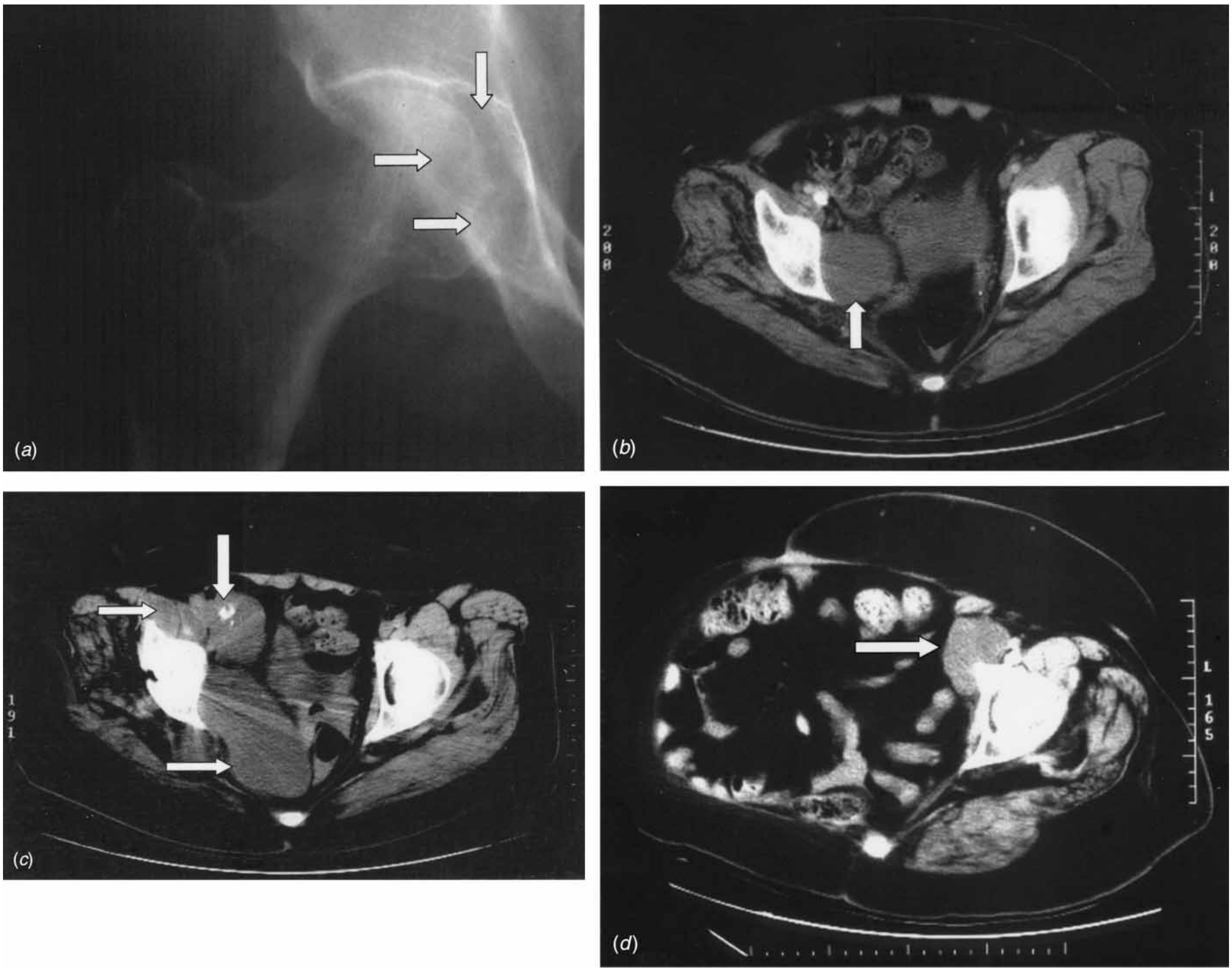

**Fig. 1.** *Sequential imaging of our case. (a) Soft tissue mass in the right hip joint. Note widening of the right hip joint (vertical arrow) and erosion of the medial aspect of the femoral head (horizontal arrow) (frontal plain film, November 1991). (b) Large 6.0-cm soft tissue mass medial to right hip, displacing the bladder (vertical arrow). Note beam hardening artifact from prosthetic hip (plain CT, January 1997). (c) New, multiple, lobulated soft tissue masses adjacent to the right hip (horizontal arrows) and the irregular 'arc and whorl' of calcification within the most medial mass (vertical arrow). Again, note beam hardening artifact from prosthetic hip (plain CT, May 1999). (d) Post-right hemipelvectomy. Note the new large 5.0-cm soft tissue mass in the left inguinal region (vertical arrow) (plain CT, May 2001).*

Unfortunately, the patient remained symptomatic. In January, 1997, her pain became unbearable. CT and ultrasound of the pelvis identified a 10 × 8-cm circular mass adjacent to the right hip, as well as a small area of calcification in the right iliac muscle (Fig. 1b). Digital rectal examination revealed a firm mass displacing the rectum to the left. The mass was excised from the medial thigh in August. Grossly, this was composed of multiple 0.3–2-cm hard nodules. Pathology once again identified a recurrence of synovial chondromatosis.

The patient's pain was unremitting, and CT now demonstrated a mass in the iliacus muscle (5 cm in diameter) and another mass next to the acetabulum of the right hip (4 cm in diameter). CT-guided biopsy of the smaller mass identified fragments of hyaline cartilaginous tissue. The masses continued to grow (Fig. 1c), and the patient underwent a right hemipelvectomy in September 1999, for pain control. Pathology now demonstrated the lesions to be a low grade chondrosarcoma arising in synovial chondromatosis (Fig. 2c). The hemipelvectomy specimen consisted of the right leg, hip joint, and portions of iliac bone and sacrum. A partly cystic mass measuring 37 × 22 × 10 cm without gross invasion of underlying bone was noted. Multiple sections showed numerous cartilaginous nodules composed of chondrocytes with mild atypia and a few binucleated cells. In some lobules, cellularity was more prominent at the periphery. No mitotic figures were present. Foci of diffuse cellularity were identified. The stroma varied in character from chondroid to myxoid. The diagnosis was supported by external consultation to a pathologist with bone and soft tissue expertise. A chest x-ray was clear.

In November 2000, the patient presented with pain in her left hip. Ultrasound and CT found a lesion anterior to the left pubic bone, and a wedge

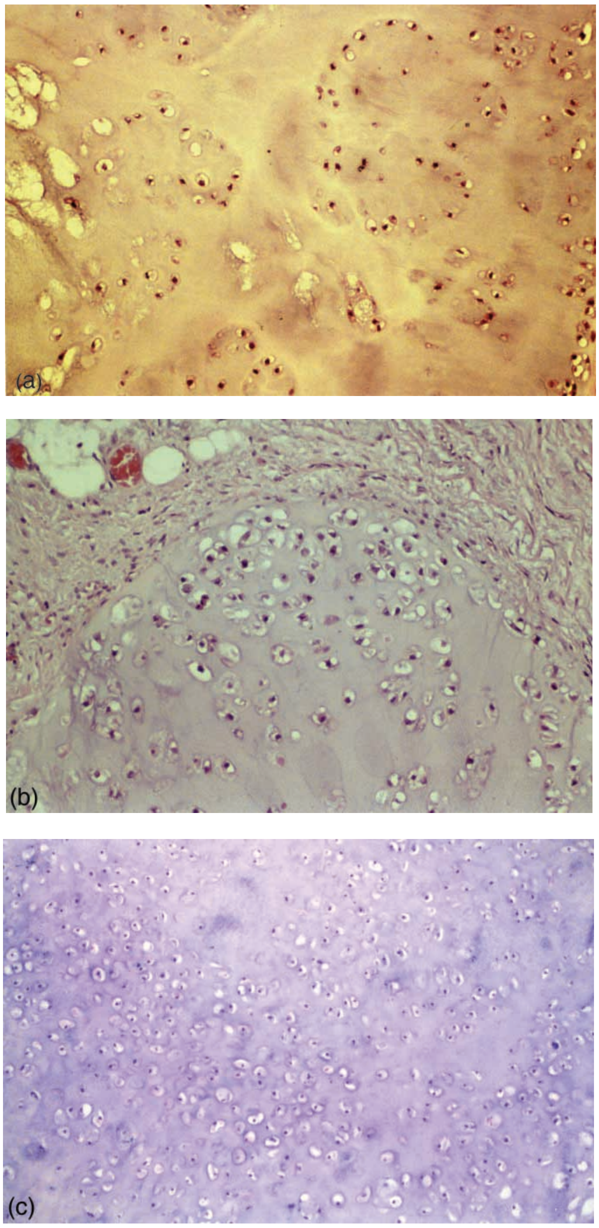

**Fig. 2.** *Sequential photomicrographs of lesions from our case, suggesting progression from synovial chondromatosis to chondrosarcoma. (a) Right hip cartilaginous clusters from 1992, showing clustering of hypocellular chondrocytes within connective tissue. The nuclei are slightly enlarged and hyperchromatic, with no significant pleomorphism. (b) Right hip cartilaginous clusters from 1994, showing a slight increase in cellularity with a relative loss of clustering architecture. There is no significant nuclear atypia. (c) Right hemipelvectomy specimen from 1999, showing (although focally) chondrocytes arranged in sheets, cellular crowding, and a focally myxoid matix, but without significant nuclear atypia.*

biopsy identified recurrent chondrosarcoma. In May 2001, pelvic CT identified four masses in total in the left groin area, suspicious for recurrent chondrosarcoma (Fig. 1d). One mass was excised, and showed recurrent low grade chondrosarcoma.

Interestingly, a second female, aged 42 years, presented in a similar fashion at this same institution during the same time period. This patient progressed from synovial chondromatosis of the left hip to chondrosarcoma within a period 11 years. Once again, numerous biopsies and resected specimens revealed a diagnosis of chondromatosis before malignancy was diagnosed. This patient refused a left hemipelvecomy for the chondrosarcoma, and also refused biopsy of lung nodules suggestive of pulmonary metastases.

### Material and methods

We (RC and SA) blindly reviewed the histology of all slides from the above two cases, mixed with six known cases of chondromatosis and five known cases of low grade (grade one and grade two) chondrosarcoma. We then investigated several immunohistochemical stains as potential discriminators of synovial chondromatosis from chondrosarcoma. The selected panel included Bcl2, Ki67, p27, p16 and p53, markers of cell survival, proliferation, and cell cycle regulators. We used the cases of known chondromatosis and low grade chondrosarcomas to serve as controls to make comparisons with the sections from our cases. All of the specimens from each surgical event were used from both of the above cases.

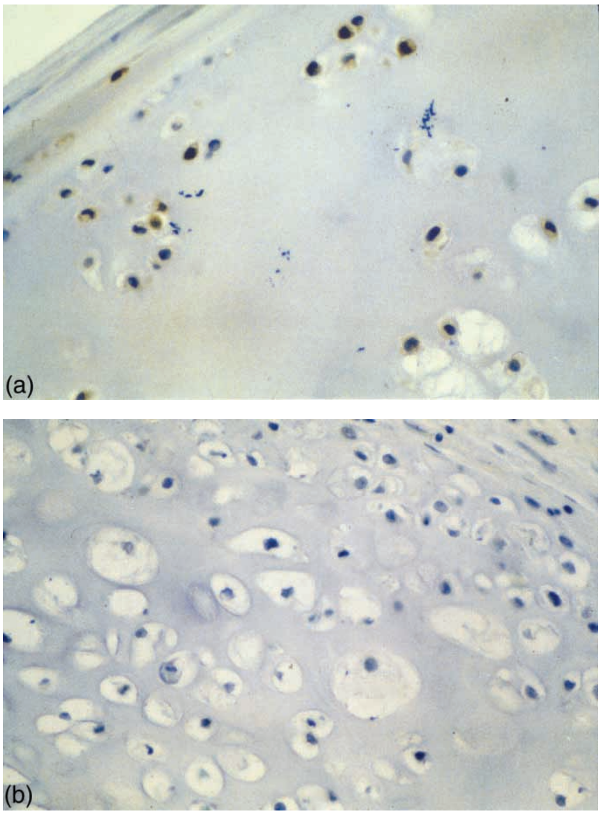

**Fig. 3.** *Immunoperoxidase staining for Bcl-2 from our case. (a) Synovial chondromatosis (1992), showing cytoplasmic positivity for Bcl-2 in the chondrocytes. (b) Chondrosarcoma (1999), showing no staining for Bcl2 in the chondrocytes.*

### Immunohistochemistry

The paraffin blocks were cut at 4–6 µm, dried overnight at 60°C, and deparaffinized in xylene. Subsequently, sections were rehydrated through graded alcohols into water. Heat-induced epitope retrieval was achieved by boiling sections in the EDTA buffer at a pH of 8.9 in the Electorolux microwave oven at 1000 W for 20 min (4 × 5 min). After boiling, sections were allowed to cool at room temperature for 20 min, rinsed thoroughly with water, and placed in Tris-buffered saline (TBS) for 5 min. After washing with TBS, sections were incubated for 30 min at room temperature with mouse anti-human antibodies against Bcl2 (clone 124, dilution 1:10, Dako Diagnostics Canada Inc, Mississauga, ON), Ki67 (MIB-1, dilution 1:75, Dako), p27 (clone SX53G8, dilution 1:20, Dako), p16 (clone F-12, dilution 1:100, Dako) and p53 (clone DO7, dilution 1:50, Dako). The immunostaining was performed using automated immunostainer (Vantana, Tucson, AZ) according to the manufacturer's instructions. Appropriate positive and negative controls were used.

### Results

On histological review of the two cases, the initial resection of masses showed the diagnostic features of chondromatosis. The resection prior to hemipelvectomy in first case and a tissue biopsy concurrent with a chest X-ray with likely metastases in second case showed focal slight increase in cellularity in nodules without a significant diffuse pattern or peripheral crowding or spindling of cells. There were scattered binucleated cells and there was mild focal atypia of chondrocytes characterized by slight nuclear enlargement and hyperchromasia, suggestive of probable early low grade chondrosarcoma. However, these features fell short of criteria to make a definitive diagnosis of chondrosarcoma. Gross and microscopic examination of the hemi-pelvectomy specimen of the first case showed nodular masses with areas of myxoid change. Microscopically, there were nodules typical of chondomatosis along with nodules showing features of chondrosarcoma: increased cellularity, peripheral spindling, myxoid stroma, and chondrocytes with mild dysplasia. No discrepancies were noted in blind review of cases with known chondromatosis and low grade chondrosarcoma.

We performed immunohistochemical stains for Bcl2 (a cell survival marker), ki67 (a cell proliferation marker), p27, p16 (cell cycle regulators) and p53 (a tumor suppressor gene) to attempt to discriminate between chondromatosis and low grade chondrosarcoma. Bcl2 was expressed at a relatively higher level in four out of five cases of control chondromatosis, and at a moderate level in one case. The Bcl2 protein was identified in chondrocytes at the periphery of the nodules. Little or no expression of Bcl2 was seen in five of six control chondrosarcomas examined; one case showed moderate expression. In slides from our two cases, Bcl2 was expressed at higher levels in early specimens diagnosed as chondromatosis; there was reduced expression in later specimens diagnosed as chondrosarcoma (Fig. 3a,b). The remaining markers showed no differences in staining between synovial chondromatosis and chondrosarcoma. Little or no immunostaining for p27 or Ki67 was identified. P16 showed moderate to marked staining in control chondromatosis cases, control chondrosarcoma cases, and slides from all specimens in both cases, and hence was not a useful marker. A rare cell showed mild to moderate staining for p53.

### Discussion

Chondrosarcoma arising in synovial chondromatosis is very rare. This case illustrates the difficulties in distinguishing recurrent chondromatosis from malignant transformation of chondromatosis. It is difficult to diagnose low grade chondrosarcoma arising in chondromatosis on histology alone, as they overlap in cyto-architectural features. In addition, progression to chondrosarcoma is focal; the findings may be easily missed with inadequate sampling of tissue for microscopy. Misinterpretation of synovial chondromatosis as a malignant lesion is a common pitfall in pathology; conversely, chondrosarcoma may be misinterpreted as a benign lesion.

Histological examination of synovial chondromatosis shows nodules of hyaline cartilage, composed of chondrocytes within synovial connective tissue. The chondrocytes are typically clustered and exhibit variations in size and nuclear chromaticity, as well as variable atypia. The degree of cellularity and nuclear atypia may equal or exceed that seen in low grade chondrosarcoma. Hence, it is very difficult to predict which chondromatosis lesions will progress to malignancy. Manivel *et al.*[4] suggest that histological features equivalent to grade two or three central chondrosarcoma must be present before diagnosing chondrosarcoma arising in synovial chondromatosis.

The histopathological distinction between these two lesions is not clear. Bertoni *et al.*[5] have suggested histological criteria for the diagnosis of malignancy: atypical chondrocytes arranged in sheets, myxoid change in the matrix, mitotic figures, crowding and spindling of nuclei at the periphery of the nodules, necrosis, and permeation of bone trabeculae. However, these criteria are unreliable; few of these changes are present in the early stages of chondrosarcoma, and are often focal. It has been suggested that the diagnosis of low grade chondrosarcoma should be made only in conjunction with unequivocal invasion beyond the joint capsule.[2]

It would be of great value to establish immunohistochemical markers that could reliably differentiate between synovial chondromatosis and low grade synovial chondrosarcoma: these two entities share clinical, radiological, and pathological features, and histological criteria are partially subjective. This distinction is important both to avoid missing a chondrosarcoma and to prevent over-diagnosis of chondrosarcoma.

In contrast to Bovee *et al.*[6] who found that Bcl2 expression was significantly higher in low grade synovial chondrosarcomas compared with synovial chondromatosis, our results demonstrate increased expression of Bcl2 in benign chondromatosis compared to synovial or central chondrosarcomas. Our study shows that a reduced or absent expression of Bcl2 may be associated with malignant transformation of chondromatosis.

In summary, distinction between chondromatosis and its progression to low grade chondrosarcoma is difficult at histological level, and must involve incorporation of clinical and radiographical data. Although preliminary, our study suggests that modulation in the immunostaining for Bcl2 may help differentiate between these two entities. More extensive study is required, including more cases and additional immunohistochemical markers.

### References

1. Helliwell TR, Ritchie DA. Tumors and tumor-like conditions of joints and juxta-articular bone. In: Hellewell TR, ed. *Pathology of Bone and Joint Neoplasms*. Philadelphia: W.B. Saunders, 1999.
2. Blokx WAM, Rasing LAJ, Beth RPH, Pruszczynski M. Late malignant transformation of biopsy proven benign synovial chondromatosis: an unexpected pitfall. *Histopathology* 2000; 36: 564–72.
3. Davis RI, Hamilton A, Biggart JD. Primary synovial chondromatosis: a clinicopathologic review and assessment of malignant potential. *Hum Pathol* 1998; 29(7): 683–8.
4. Manivel JC, Dehner LP, Thompson R. Case report 460: synovial chondrosarcoma of left knee. *Skeletal Radiol* 1988: 17(1): 66–71.
5. Bertoni F, Unni KK, Beabout JW, *et al.* Chondrosarcomas of the synovium. *Cancer* 1991; 67: 155–62.
6. Bovee JVMG, van den Broek JCM, Cleton-Jansen AM, Hogendoorn CW. Up-regulation of PTHrP and Bcl-2 expression characterizes the progression of osteochondroma towards peripheral chondrosarcoma and is a late event in central chondrosarcoma. *Lab Invest* 2000; 80: 1925–33.
